# Screening of the Chemical Composition and Identification of Hyaluronic Acid in Food Supplements by Fractionation and Fourier-Transform Infrared Spectroscopy

**DOI:** 10.3390/polym13224002

**Published:** 2021-11-19

**Authors:** Tamilla Mirzayeva, Jana Čopíková, František Kvasnička, Roman Bleha, Andriy Synytsya

**Affiliations:** 1 Department of Carbohydrates and Cereals, University of Chemistry and Technology, Technická 5 Dejvice, 166 28 Prague, Czech Republic; copikovj@vscht.cz (J.Č.); blehar@vscht.cz (R.B.); sinicaa@vscht.cz (A.S.); 2 Department of Food Preservation, University of Chemistry and Technology, Technická 5 Dejvice, 166 28 Prague, Czech Republic; Frantisek.Kvasnicka@vscht.cz

**Keywords:** hyaluronic acid, determination, food supplements, Fourier-transform infrared spectroscopy

## Abstract

Hyaluronic acid, together with collagen, vitamins or plant extracts, is a part of many cosmetic and food preparations. For example, this polysaccharide is used in formulation of many food supplements due to its protective effects on human health. In this work, the screening of the chemical composition of three chosen dietary supplements (powder, tablets and capsules) containing hyaluronic acid was carried out using Fourier-transform infrared spectroscopy. Because of the low amount of analyte in all these samples, it was isolated or concentrated prior to the analysis using a suitable sequential fractionation protocol. Individual isolation procedures were established for each sample based on their declared composition. Firstly, the major components such as collagen or vitamins were removed to obtain polysaccharide fractions by the enzymatic treatment and/or washing out with the appropriate solvents. In some cases, the water insoluble part was removed from the rest dissolved in water. Then, hyaluronic acid was precipitated with copper(II) cations and thus separated from the other polysaccharides. Finally, the analyte was identified in the enriched fractions by the characteristic vibrational bands. The amount of hyaluronic acid in the purified fractions was determined in three ways: gravimetrically, spectrophotometrically, and using isotachophoresis. The combination of the appropriate preparative and analytical steps led to the successful evaluation of chemical composition, finding and quantification of hyaluronic acid in all the studied samples.

## 1. Introduction

Hyaluronic acid is an acidic polysaccharide of animal and bacterial origin that serves various physiological purposes, for example, in connective tissues of vertebrates and bacterial capsules [1,2,3,4]. It is an alternating co-polymer of 1,4-linked *β*-d-glucuronic acid and 1,3-linked *N*-acetyl-*β*-d-glucosamine. Hyaluronic acid is the only non-sulfated glycosaminoglycan with unique physicochemical and biological properties [5]. It is a highly hygroscopic polymer that can hold water molecules and create a gel-like environment [6,7,8]. Even at low concentrations in an aqueous medium, hyaluronic acid and its salts, hyaluronates, behave like viscoelastic systems [9,10,11,12]. A number of charged and polar groups allow hyaluronic acid to hold water molecules and participate in many biologically important polar interactions. On the other hand, regularly repeating *N*-acetyl groups promote interaction with cell membranes and hydrophobic regions of membrane proteins, which is important for ensuring cell motility [13]. Thus, the combination of hydrophilic and hydrophobic characteristics is an integral part of the basic structure of this polysaccharide [14,15,16].

Hyaluronic acid is involved in protective and other physiological processes, including healing of wound and burns [17,18,19,20,21], tissue regeneration [22,23], cell differentiation, morphogenesis, angiogenesis, and inflammation [23,24,25]. Biocompatibility and high affinity for water make it possible to use hyaluronic acid in various fields of medicine. Natural hyaluronic acid itself or healing systems containing this polysaccharide are used in surgery, pharmacology, ophthalmology, dermatology and cosmetology [26,27,28,29]. It is a component of synovial fluid substitutes as a medium for ocular surgery, preservation and cell transfer. This polysaccharide has been used in various nutritional supplements and cosmetics.

Determination of hyaluronic acid in food supplements is difficult due to the low content of this analyte, as well as the presence of many other components such as proteins (collagen), other polysaccharides, vitamins or plant extracts. Moreover, these substances are often present in larger quantities than the target compound, which further complicates the analysis. Hyaluronic acid has been determined as intact polymeric chains in food supplements and medicinal preparations by separation methods including capillary zone electrophoresis (CZE), isotachophoresis (ITP) and high-performance liquid chromatography (HPLC) [30,31,32,33,34]. Another approach is based on a combination of complete or partial hydrolysis using mineral acids or enzymes, followed by quantitative analysis of the hydrolysate by photometry, separation or electromigration methods [35,36,37]. Both approaches have a number of disadvantages associated primarily with large differences in molecular weight or with incomplete hydrolysis of hyaluronic acid macromolecules. In addition, the presence of various substances in the samples can interfere with the determination, which means that they must be removed before analysis. This presupposes an individual approach to each sample, depending on their composition declared by the manufacturer. As an alternative approach, we can offer sequential purification of the sample using suitable extraction and precipitation steps, and the composition and purity of the resulting fractions can be assessed using Fourier-transform infrared (FT-IR) spectroscopy as a fast, structure-sensitive and non-invasive method that has been used in structural and conformational analysis of hyaluronic acid, hyaluronates and derived oligomers [38,39,40,41,42,43,44]. In addition to this, FT-IR spectroscopy can be used as screening method to evaluate the composition of food supplements and thus detect not only hyaluronic acid but also the other organic and some inorganic components, which are not transparent in the middle infrared region.

The disadvantage of FT-IR spectroscopy compared to spectrophotometry and separation methods such as HPLC or CZE is its low sensitivity, so it is well suited only for the determination of major components. Therefore, in order to detect hyaluronic acid in dietary supplements, it is necessary to concentrate it through suitable purification steps. The water-insoluble part can be easily separated from the aqueous solution of the remaining compounds, small molecules can be washed out using suitable solvents, and the proteins must be pre-hydrolyzed with proteases, and then the resulting peptides must be removed in the same way as other small molecules. A difficult situation can arise if, as a result of all the above stages, hyaluronic acid remains in a mixture with other polysaccharides that can be added together with it to the formulation, for example, with starch or starch modification products. In this case, precipitation with metal counter cations can be used to separate hyaluronic acid from neutral polysaccharides.

Multivalent metal cations are known as effective cross-linkers for biopolymers including anionic polysaccharides [45]. When treated with such metal cations, polysaccharide gels and precipitates can form. Precipitation by metal cations has been used for preparative and analytical purposes as a method for the rapid separation of macromolecules depending on their charge. For example, precipitation with copper(II) cations has been used for the purification of pectins, anionic plant cell wall polysaccharides [46,47,48,49]. This approach has been also used for determination of pectin content by photometry [50]. The precipitation of pectins by metal cations is based on the selective formation of insoluble complexes. Compared to precipitation with ethanol, the use of metal counter cations in the precipitation of apple pectins led to an increase in galacturonic acid content [46]. Metal precipitation avoids co-precipitation of other polysaccharides, which is observed in the ethanol precipitation [47]. Copper(II) cations selectively bind anionic regions of pectin chains, thereby separating pectic polysaccharides from oligosaccharides and proteins [48]. Consequently, counter cations have a certain affinity for negatively charged structural regions of macromolecules, thereby selectively precipitating polyuronic acids, while neutral polysaccharides remain in solution. Similarly to pectins, copper(II) cations can be used for the precipitation of other polyuronides including hyaluronates. However, unlike pectins, the precipitation of hyaluronic acid with copper(II) cations has not been sufficiently studied and widely used, although copper(II) hyaluronates have been prepared and described [51,52,53,54,55]. Copper(II) cations are coordinated with hyaluronate through the carboxylate group and anomeric oxygen of the *β*-d-glucuronic acid units, while the *N*-acetamide group of the *N*-acetyl-*β*-d-glucosamine units is not involved in coordination. Therefore, as in the case of pectins, copper(II) cations are able to crosslink hyaluronic acid macromolecules through carboxylate groups and therefore precipitate copper(II) hyaluronate from a solution in which neutral macromolecules remain. Precipitation of copper(II) hyaluronate occurs under neutral and weak alkaline conditions, while under weakly acidic conditions, the complex is stable in solution [51], possibly due to incomplete dissociation of carboxylic groups [49].

This work is devoted to the screening analysis of chemical composition, identification and quantification of hyaluronic acid in three dietary supplements of different dosage forms (powder doze, tablet and capsule) and composition declared by manufacturers using a combination of suitable purification steps, including precipitation with copper(II) cations, and FT-IR spectroscopy of the fractions obtained. For each sample, individual isolation procedures for analyte enrichment were chosen in relation to sample composition and analyte content in raw material. Quantification was carried out gravimetrically by the mass of the final fractions identified by FT-IR as hyaluronic acid, as well as by spectrophotometry and isotachophoresis. This approach is suitable for the detailed characterization of food supplements and similar samples containing hyaluronic acid together with an excess of other components (collagen, polysaccharides, vitamins, etc.).

## 2. Materials and Methods

### 2.1. Samples and Chemicals

The purpose of the study was to identify the hyaluronic acid in three samples of dietary supplements with different composition obtained from the manufacturers and/or from the domestic market (Table 1). Expected amount of hyaluronic acid in these supplements was in the range of ~0.8–5.9% *w*/*w*.

Following chemicals were used in this work:

- Hyaluronic acid, glycine, 2-hydroxyethylcellulose, pepsin, copper(II) chloride dihydrate (Sigma-Aldrich, Saint Louis, MO, USA)
- Citric acid (Lach-Ner Ltd., Neratovice, Czech Republic)
- Ethanol 96%, ethyl acetate, hexane 99%, sodium hydroxide, hydrochloric acid, sodium carbonate, sodium hydrogen carbonate (PENTA s.r.o., Prague, Czech Republic)
- Potassium bromide for IR spectroscopy (Merck KGaA, Darmstadt, Germany)

A carbonate-bicarbonate buffer was prepared by dissolving 129 g of Na_2_CO_3_ and 50 g of NaHCO_3_ in 1 L of distilled water [56]. All chemicals were of analytical grade. Deionized water of Milli-Q quality (electrical resistivity 18.2 MΩ·cm) was used for the electrolyte, standard and sample preparations.

### 2.2. Preparative Procedures

**Figure 1:**
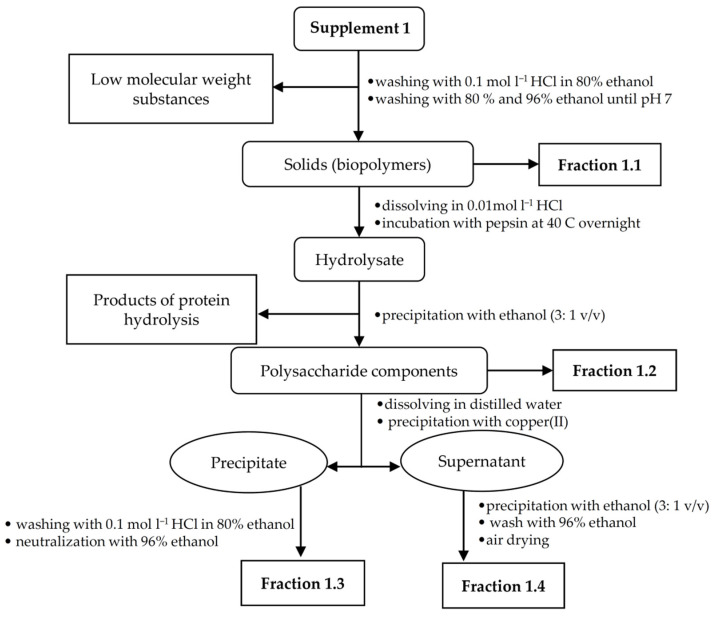
Isolation scheme for supplement 1.

**Figure 2:**
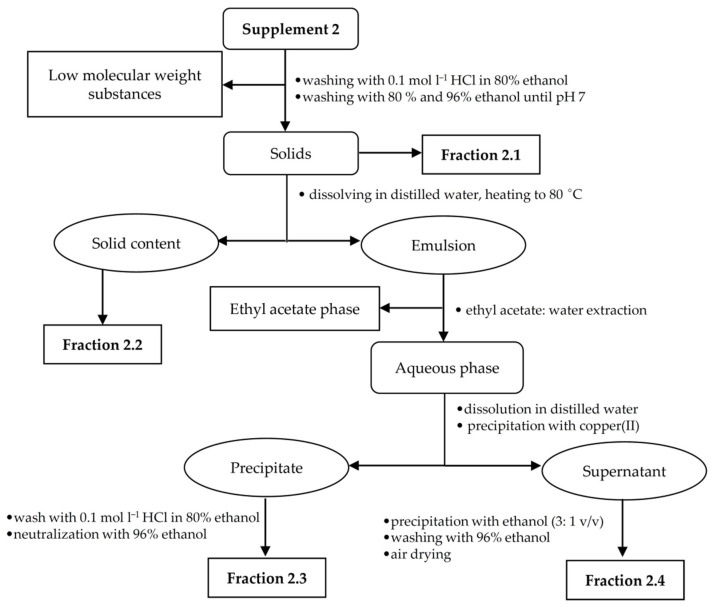
Isolation scheme for supplement 2.

**Figure 3:**
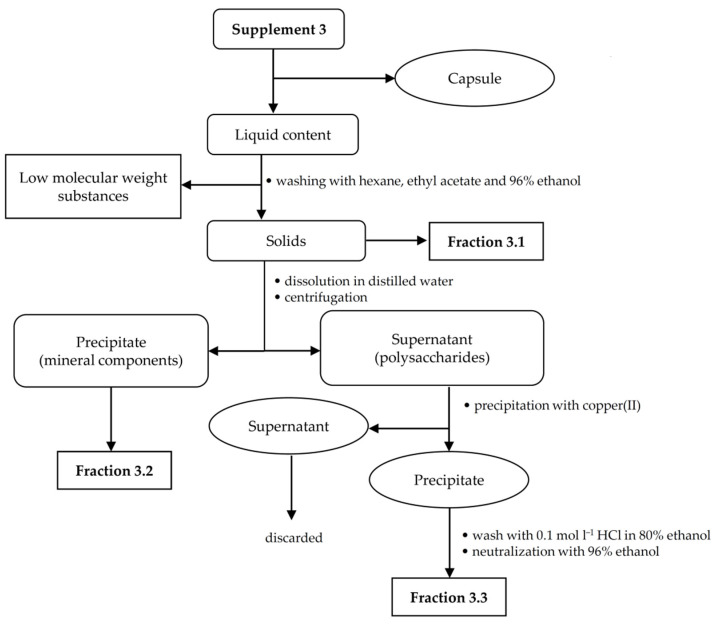
Isolation scheme for supplement 3.

Due to the low amount of the analyte in all food supplements, the appropriate isolation procedures were made to obtain fractions rich of hyaluronic acid. For each supplement sample, a specific isolation procedure was developed based on a combination of extraction and precipitation steps. An individual hyaluronic acid isolation procedure was chosen for each sample in relation to the sample composition and analyte content. The presence of target polysaccharide in the enriched fractions was confirmed using FT-IR spectroscopy. Isolation procedures for supplements 1–3 are illustrated in Figure 1, Figure 2 and Figure 3.

#### 2.2.1. Fractionation of Supplement 1

The powder sample was weighed (13.2 g) and successively washed with 96% ethanol, acidified ethanol (0.1 mol L^−1^ HCl in 80% ethanol) and then again with 96% ethanol. The ethanolic suspensions were stirred on a magnetic stirrer for 30 min. Then, the mixture was filtered through filter paper and the solids assigned as fraction 1.1 were dried on a watch glass. The dried solids were dissolved in 50 mL of 0.01 mol L^−1^ hydrochloric acid, and 4 g of pepsin was added to the solution. Next, the solution was stirred for 30 min on a magnetic stirrer C-MAG HS7 (IKA-Werke, Staufen, Germany), and after dissolving the enzyme; it was placed in a thermostat BT 120 (LABsystem Praha s.r.o., Prague, Czech Republic) at 40 °C overnight. The enzyme was then denatured by heating the solution at 90 °C for 10–15 min on magnetic stirrer and filtrated. The solution was cooled and solids were precipitated with an excess of 96% ethanol (3: 1 *v*/*v*) and then filtered off. The resulting precipitate (0.65 g) was air dried and designated as fraction 1.2. The dried precipitate was ground in a powder mortar and then dissolved in 20 mL of distilled water. A small amount of copper(II) chloride was added with stirring. The pH value was adjusted to 7.0 by adding a small volume of 0.1 mol L^−1^ sodium hydroxide solution. The resulting precipitate, designated as fraction 1.3, was centrifuged and washed with acidified ethanol to remove copper(II) cations that were detected by visual decolonization of the precipitate. Finally, the precipitate was washed with 96% ethanol to pH ~7.0. The supernatant was precipitated with excess ethanol and the resulting precipitate, i.e., fraction 1.4, was washed in the same way as fraction 1.3.

#### 2.2.2. Fractionation of Supplement 2

The sample (30 tablets) was weighed and then washed with 96% ethanol, acidified ethanol and then again with 96% ethanol yielding fraction 2.1. Cellulose was separated from the other polysaccharides by suspending this fraction in 150 mL of distilled water and heating to 80 °C. The mixture was filtered through filter paper. The solids, assigned as fraction 2.2, contained cellulose, which is insoluble in water, while the filtrate contained water-soluble polysaccharides including hyaluronic acid. The filtrate was orange due to the presence of β-carotene, which was then removed by washing with ethyl acetate, and a small amount of copper(II) chloride was added to the clear solution with stirring. The pH was adjusted to 7.0 by adding of sodium hydroxide. The resulting precipitate, i.e., fraction 2.3, containing hyaluronic acid, was washed with acidified ethanol and further with 96% ethanol to remove copper(II) cations from the sample. The neutral polysaccharides were precipitated from supernatant with ethanol excess, and the resulting precipitate, i.e., fraction 2.4, was washed with 96% ethanol.

#### 2.2.3. Fractionation of Supplement 3

Firstly, the shells of 10 capsules were removed, and the internal liquid content (emulsion) was poured onto a watch glass. Then, the emulsion (3.4 g) was decomposed and decolorized by successive washing with hexane, ethyl acetate and 96% ethanol. As a result, insoluble powdered fraction 3.1 was obtained. The presence of insoluble minerals such as zinc(II) oxide has been declared by the manufacturer (see Table 1). To remove these mineral components, the obtained powder was dissolved in a small amount of distilled water and further centrifuged using Sigma 2-16K centrifuge (Sigma-Aldrich, Saint Louis, MO, USA). The precipitate, assigned as fraction 3.2, was dried on a watch glass. The supernatant, contained the expected polysaccharide components, was used for further fractionation. A small amount of copper(II) chloride was added with stirring and the pH was adjusted to 7.0 by adding 0.1 mol L^−1^ sodium hydroxide. The precipitate formed was washed with acidified ethanol and further with 96% ethanol to remove copper(II) cations and then centrifuged. These solids containing hyaluronic acid were assigned as fraction 3.3. The supernatant was treated with excess ethanol, but no precipitate was obtained; therefore, the sample did not contain neutral polysaccharides.

### 2.3. FT-IR Spectroscopy

FT-IR spectra (400–4000 cm^−1^) of the supplements and isolated fractions were recorded in KBr pellets by Nicolet 6700 FT-IR spectrometer using Omnic 8.0 software (ThermoFisher Scientific, Waltham, MA, USA); 64 scans were accumulated with a spectral resolution of 2.0 cm^−1^. The spectra were smoothed by 11 points, baseline corrected, and exported in ASCII format to Origin 6.0 (Microcal Origin, Northampton, MA, USA) software for preparation of graphs. The position of shoulders was determined using the 2nd derivative algorithm.

### 2.4. Isotachophoresis

Isotachophoretic measurement of hyaluronic acid in partially purified dietary supplements was recorded using a hand-held column electrophoretic analyzer EA 101 with contact conductivity detectors and a UV detector operating at 254 nm (Villa-Labeco, Spišská Nová Ves, Slovak Republic) [57,58]. The separation section of the analyzer consisted of a pre-separating FEP capillary (90 mm × 0.8 mm i.d) in conjunction with an analytical FEP capillary (90 mm × 0.3 mm i.d) and was controlled by a PC and ITPPro32 software (KasComp Ltd., Bratislava, Slovak Republic). The device works with a hydrodynamically closed separation system, i.e., the electroosmotic flow is suppressed. For anionic analysis, a mixture of 5 mmol L^−1^ HCl + 10 mmol L^−1^ glycine + 0.05% 2-hydroxyethylcellulose was used as the main electrolyte, and the 10 mmol L^−1^ citric acid solution served as the terminal electrolyte. The method of adding an internal standard of hyaluronic acid was used. The analyte was detected using a conductivity detectorof EA 101 analyzer. The results were presented in the form of isotachopherograms.

### 2.5. Photometry

The content of hyaluronic acid in dietary supplements was determined by photometry according to the method which has been proposed for the determination of pectin [50], with some modifications. The method was adapted for analysis of hyaluronic acid and is based on the precipitation of copper(II) hyaluronates by the treatment with copper(II) chloride and estimation of the remaining content of copper(II) ions as a complex with carbonate. One and a half milliliters of the filtrate after precipitation of copper(II) hyaluronate was mixed with 2 mL of the carbonate buffer solution, and the absorbance of the mixtures was recorded at 712 nm with a UV-vis spectrophotometer Specord 50 Plus (Analytic Jena, Jena, Germany). Standard aqueous solutions of copper(II) chloride (2–100 mmol L^−1^) in mixture with carbonate buffer (1: 4 *v*/*v*) were used for calibration. For quantitative determination, a standard solution of hyaluronic acid was used.

## 3. Results and Discussion

### 3.1. Description of the Isolated Fractions

Because of the low amount of analyte in the samples, it was decided to isolate hyaluronic acid from food supplements and subsequently confirm its presence in the enriched fractions by FT-IR spectroscopy. The first step was the removal of major components such as collagen and low-molecular substances, in particular various vitamins, to obtain polysaccharide fractions for further purification. Enzymatic hydrolysis by pepsin led to removal of already partially hydrolyzed collagen. The low-molecular components were washed out by suitable organic solvents. Then, hyaluronic acid was separated from the other polysaccharides present in the fractions, including cellulose and maltodextrins. Soluble polysaccharides, including hyaluronic acid, were separated from cellulose by dissolving in water. Finally, hyaluronic acid was precipitated with copper(II) cations and thus separated from other maltodextrins. The presence of hyaluronic acid was confirmed by FT-IR spectroscopy based on the assignment of the band’s characteristic for this polysaccharide and comparison with the literature. The combination of the described preparative and analytical steps and methods led to the successful finding of hyaluronic acid in all the studied samples.

The chosen procedure for the isolation of hyaluronic acid from the starting material should meet two basic requirements. First, the process should be as efficient as possible, along with minimal losses during the isolation process. If the sample contains low molecular weight hyaluronic acid or its fragments (oligosaccharides), which can dissolve in dilute aqueous ethanol, there is a possibility of analyte loss. To prevent this, precipitation with excess ethanol should be replaced by a combination of dialysis through suitable membranes and lyophilization. Second, there should be no structural degradation of the polysaccharide during the isolation process. It is essential that the sample does not decompose during operation by chemicals or enzymes used to remove unwanted components.

Table 2 shows the yields of the individual fractions (% *w*/*w*) of each sample and their expected composition. Supplement 1 contained mainly biopolymer components (collagen, maltodextrins) in addition to hyaluronic acid. Enzymatic hydrolysis with pepsin was chosen to remove collagen. Supplements 2 and 3 contained a large amount of low molecular weight substances that had to be removed first.

The next step was to separate the individual polysaccharides based on their solubility or ability to form complexes with copper(II) cations. Thus, hyaluronic acid was separated from cellulose and maltodextrins. Fractions 1.3 and 2.3, which allegedly contained hyaluronic acid, were determined in the case of supplement 2 to less than a tenth of a percent. On the contrary, the respective fraction 3.3 of supplement 3 (capsule content) had a content in the order of units of percent, which corresponds to a larger amount of this polysaccharide in the sample, as declared by the manufacturer (see Table 1). It was also found based on the comparison of FT-IR spectra of the above fractions that the largest amount of analyte was present in fraction 3.3 of supplement 3, which is an advantage for proving its presence.

### 3.2. FT-IR Spectra

#### 3.2.1. Raw Supplements

**Figure 4:**
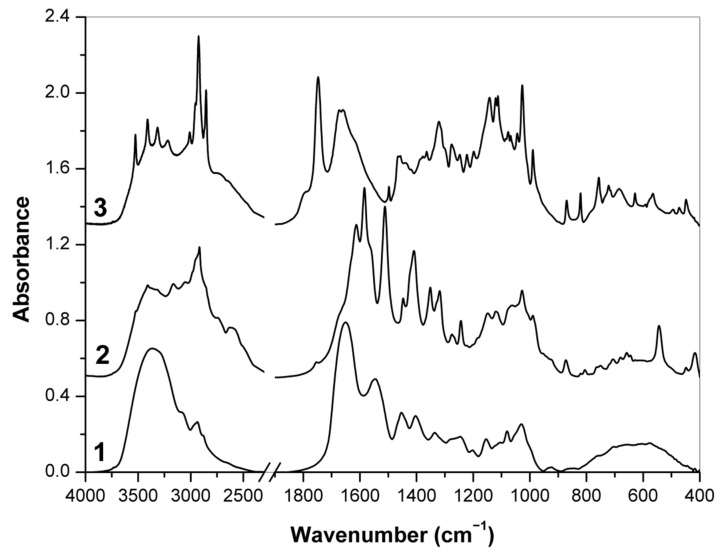
FT-IR spectra of supplements 1, 2 and 3 (in the latter case, capsule content).

The FT-IR spectra of food supplements 1, 2 and 3 where the presence of hyaluronic acid was declared are shown in Figure 4. The FT-IR spectrum of supplement 1 has wide, distinct bands of biopolymer components and their derivatives—partially hydrolyzed collagen and maltodextrins. Two intense bands of amide vibrations at 1650 and 1545 cm^−1^ indicate the presence of proteins [59,60], i.e., modified collagen, while several less pronounced bands at 1154, 1080 and 1030 cm^−1^ originated mainly from COC, CO and CC stretching vibrations in carbohydrates which are typical for starch and its oligomers dextrins [61,62]. By contrast, there are much narrower bands of low molecular weight components in the spectra of supplements 2 and 3. In both cases, the most of intense bands corresponded to vibrations of the major components, i.e., l-methionine (~46 % *w*/*w*) and l-ascorbic acid (~46% *w*/*w*), respectively, as it has been declared by manufacturers (Table 1). Indeed, the intense bands of l-methionine at 2917 cm^−1^ (CH_2_ symmetric stretching), 1613 and 1512 cm^−1^ (NH_3_^+^ bending), 1584 and 1409 cm^−1^ (COO– stretching), 1447 and 1352 cm^−1^ (CH_2_ bending), 1318 cm^−1^ (SCH and CH_3_ bending), 1275 and 1243 cm^−1^ (CCH and SCH bending), 1027 (CC stretching), 543 and 418 cm^−1^ (CCO and CCN bending) as well as many other bands of this amino acid were found in the FT-IR spectrum of supplement 2 [63,64]. Similarly, the bands of l-ascorbic acid at 3525–3215 cm^−1^ (OH stretching), 1673 cm^−1^ (C=C stretching), 1660, 1457 and 1246 cm^−1^ (CH and CH_2_ deformation modes), 1364, 1321 and 1276 cm^−1^ (in-plane ring deformation), 1142–821 cm^−1^ (CO and CC stretching), 756 (in-plane OH twisting), 722 and 685 cm^−1^ (C=O deformation modes) and skeletal ring vibrations at 629–448 cm^−1^ [65] were found to be the most intense in the FT-IR spectrum of supplement 3. In addition, the IR band around 1748 cm^−1^ indicates stretching C=O vibrations of organic acids and esters, and together with other bands at 2925, 2855, 1466 and 721 cm^−1^ (vibration of CH_2_ groups) confirmed the presence of fats [66]. In contrast, the broad bands in the region of 3600–3200 cm^−1^ were assigned to the stretching vibrations of OH and NH bonds. However, characteristic bands of hyaluronic acid were not pronounced in FT-IR spectra of all raw supplements 1–3 because of low amounts of this polysaccharide.

#### 3.2.2. Fractionation of Supplement 1

**Figure 5:**
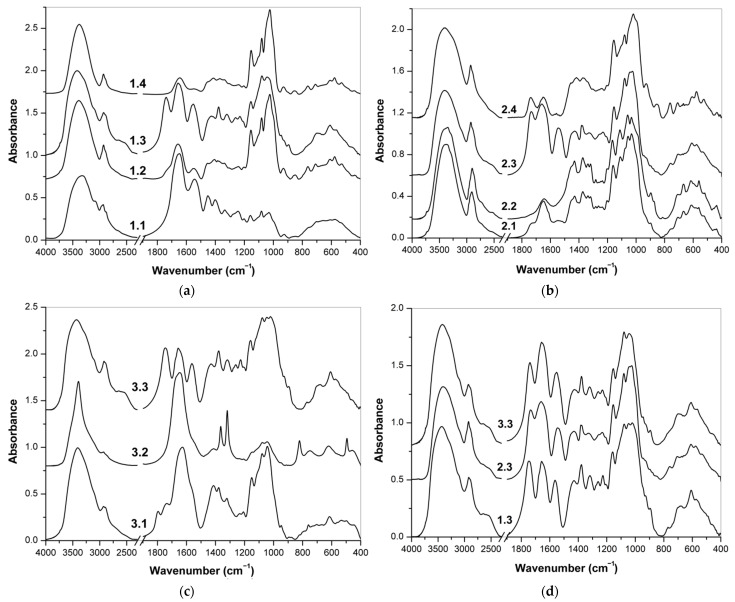
FT-IR spectra of the fractions obtained from: (**a**) supplement 1; (**b**) supplement 2; (**c**) supplement 3; (**d**) FT-IR spectra of the fractions 1.3, 2.3 and 3.3 with an expected high content of hyaluronic acid obtained from supplements 1, 2 and 3, respectively.

The FT-IR spectra of fractions 1.1–1.4 isolated from food supplement 1 are shown in Figure 5a. The spectrum of fraction 1.1, obtained by washing with acidified ethanol, did not differ significantly from the spectrum of the initial supplement, which indicates that the sample contained few low-molecular-weight substances. In the spectrum of fraction 1.2, after enzymatic removal of proteins, the protein bands at 1800–1500 cm^−1^ (amide I and amide II vibrations) significantly decreased [59,60]. In contrast, the polysaccharide bands in the 1200–950 cm^−1^ region were much more intense. These results confirm that as a result of enzymatic hydrolysis, the collagen content decreased, and at the same time, the proportion of polysaccharides, mainly maltodextrins, increased. In the FT-IR spectrum of fraction 1.3 obtained by precipitation with copper(II), bands at 1738, 1617 and 1560 cm^−1^ indicate the presence of carboxyl and amide groups of hyaluronic acid [39,40]. On the contrary, fraction 1.4 contained mainly maltodextrins, which do not form copper (II) complexes in a neutral medium, which is confirmed by several strong bands at 1154, 1080 and 1030 cm^−1^ (stretching vibrations of CO and CC), as well as weaker bands of skeletal vibrations in the region of 930–445 cm^−1^ [61,62].

#### 3.2.3. Fractionation of Supplement 2

The FT-IR spectra of fractions 2.1–2.4 isolated from additive 2 are shown in Figure 5b. The spectrum of fraction 2.1 after washing with acidified ethanol showed significant differences from the spectrum of the original sample due to the removal of l-methionine and other low molecular weight substances. The spectrum contains a number of bands at 3346, 2902, 1432, 1373, 1337, 1319, 1165, 1113, 1059, 1032, 897, 710, 668, 617, 560, 519 and 436 cm^−1^ characteristics of cellulose [67,68]. This polysaccharide was probably not added to the preparation as a pure substance but apparently was included in the plant extracts. The FT-IR spectrum of the water-insoluble part 2.2 showed much more pronounced cellulose bands. This fraction was defined as almost pure cellulose, separated from water-soluble polysaccharides, including hyaluronic acid. Two more fractions were formed by precipitation of copper(II) complexes from the water-soluble part. Hyaluronic acid was observed in precipitate 2.3 based on characteristic IR bands. Finally, the FT-IR spectrum of supernatant 2.4 showed intense bands of starch originating from plant extracts [61] and less pronounced bands of carboxyl and amide groups compared to the previous fraction. This means that there was an incomplete separation of polysaccharides and proteins with a larger proportion of hyaluronic acid entering fraction 2.3.

#### 3.2.4. Fractionation of Supplement 3

The last supplement was in the form of gelatin capsules with a liquid mixture of active ingredients inside. The liquid contents of the capsules were isolated from the shells and thus separated from the solid gelatin. The obtained FT-IR spectra of fractions 3.1, 3.2 and 3.3 are shown in Figure 5c. The spectrum of fraction 3.1 no longer contained bands of l-ascorbic acid, fats and other low molecular weight components, and various bands in the areas 1800–1500 cm^−1^ and 1200-950 cm^−1^ indicated the presence of hyaluronic acid, but there are a number of other bands. The water-insoluble part of 3.2 had a very different FT-IR spectrum. This fraction probably contained insoluble inorganic substances (metal oxides, etc.). Finally, fraction 3.3 was precipitated with copper(II) cations and then purified. This fraction contained mainly hyaluronic acid, which was confirmed by a number of characteristic bands, including vibrations of amide and carboxyl groups (see Table 3).

### 3.3. Detection and Quantification of Hyaluronic Acid

The FT-IR spectra of fractions 1.3, 2.3 and 3.3 derived from supplements 1–3 and allegedly containing hyaluronic acid are shown in Figure 5d. A number of characteristic bands summarized and assigned in Table 3 confirmed the presence of this polysaccharide in acidic form. All these fractions evidently contained this polysaccharide as the major part, and the characteristic bands of hyaluronic acid were sufficiently prominent for identification. However, fraction 2.3 also contained some proteins because the amide I and amide II bands are evidently much more intense than the bands of C=O stretching vibration in carboxylic groups at 1732 cm^−1^. In addition, the band of amide II vibration is shifted to 1545 cm^−1^, which is typical for proteins, so as the positions of some other amide bands. Due to the importance of the amide and carboxyl groups of hyaluronic acid, the characteristic IR bands of these groups are key markers for the identification of this polysaccharide [39]. Bands at 3275–3277, 3094–3098, 1657, 1552–1563 and 1318–1320 cm^−1^ are indicative for the amide groups in *N*-acetyl-*β*-d-glucosamine units, and the bands at ~2660, 1732–1745, 1260 and 923–930 cm^−1^ are assigned to the vibrations of the carboxylic groups in *β*-d-glucuronic acid units [38,39,40]. In the case of salts (hyaluronates), these bands of carboxylic vibrations do not occur in the spectra; instead, two bands around 1618 and 1411 cm^−1^ corresponding to the antisymmetric and symmetric valence vibration COO– are present [39,40].

**Figure 6:**
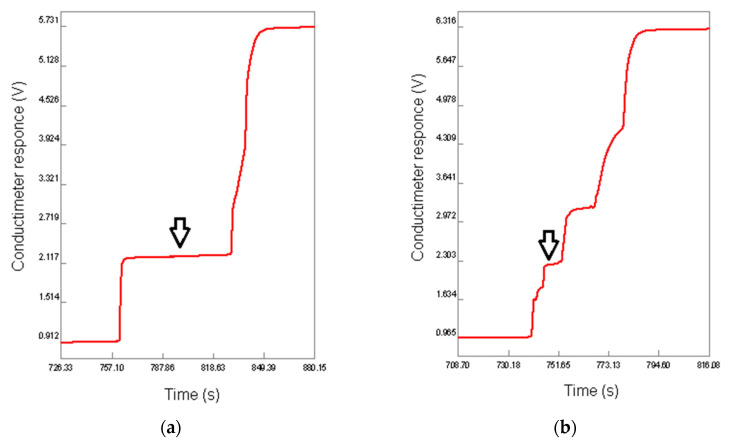
Isotachopherograms of the standard solution of hyaluronic acid (**a**) and supplement 1 (**b**). Arrow indicates the analyte zone.

**Figure 7:**
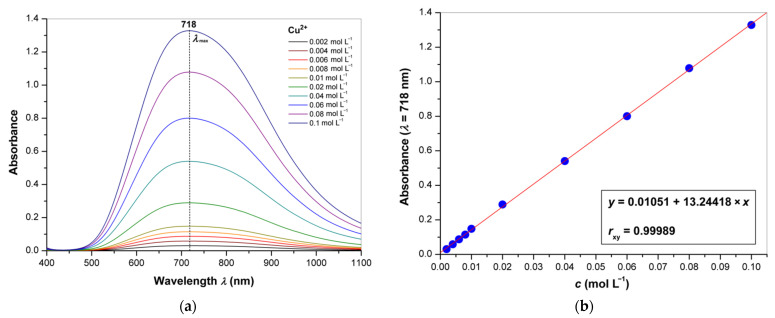
(**a**) Absorption spectra of copper(II) chloride (0.002–0.1 mol L^−1^) dissolved in the carbonate buffer; (**b**) calibration curve for determination of copper(II).

Since fractions 1.3, 2.3 and 3.3 were identified as hyaluronic acid by FT-IR, their relative masses were used to quantify this analyte gravimetrically in supplements 1, 2 and 3, respectively. Moreover, the level of hyaluronic acids in the supplements was determined by spectrophotometry and isotachophoresis. The isotachopherograms of the standard solution of hyaluronic acid and the purified fraction of supplement 1 are shown in Figure 6a,b; the arrow indicates the zone of hyaluronic acid. The height of the zone corresponds to a certain anionic compound (analyte) in accordance with its mobility in an electric field, and the length of the zone is proportional to the concentration of this analyte. The absorption spectra of the copper(II) chloride solutions and the calibration curve for quantification of copper(II) cations are represented in Figure 7a,b. The wavelength of absorption maximum at 718 nm was used for the analysis; this value is much closed to that in the literature (712 nm) [50]. The obtained results are summarized in Table 4 in comparison with the corresponding declared values represented in % *w*/*w*. It can be seen from this table that the values obtained by the three methods coincide with each other and are quite close to the declared values. For the spectrophotometry, the relative standard deviations (RSD) in three measurements were less than 3%, indicating good reliability for determination of hyaluronic acid in food supplements. These values were similar to those obtained previously for the determination of pectin [50] and for the earlier reported isotachophoretic method [57,58]. In any case, all of the analytical methods used in this work can be recommended for the analysis of food supplements containing hyaluronic acid, but each of them has its own advantages and disadvantages, which are summarized in Table 5. Besides, effective fractionation allows for removal of impurities that interfere with the analysis, for example, proteins and their fragments (peptides) that can be precipitated by copper(II) cations together with hyaluronic acid. Partially, FTIR and isotachophoresis are of interest as polyanalytical methods that can be used to analyze many other components, not only hyaluronic acid. FTIR is able to evaluate the composition of individual fractions and confirm or deny the presence of the declared substances. For example, in the current study, the presence of undeclared cellulose was proven for the water-insoluble fraction 2.2 isolated from supplement 2. Both methods also allow for the determination of other glycosaminoglycans [43,57,58,69,70,71], which is of particular interest since chondroitin sulfate is often used in dietary supplements to support cartilage along with collagen and hyaluronic acid. On the other hand, the spectrophotometric method for the determination of copper(II) as the carbonate complex is quite sensitive and allows determination over a wide range of concentrations. However, care should be taken to ensure that the pH value is optimal and does not lead to alkalization and precipitation of copper(II) hydroxide. On the contrary, upon acidification, the copper(II) complex with hyaluronic acid may remain in solution due to insufficient ionization of polysaccharide macromolecules [49,51,52,53]. Finally, although gravimetry is not structure-sensitive, FT-IR can be used to determine if the precipitate formed is actually hyaluronic acid, with an assessment of possible impurities.

## 4. Conclusions

The aim of this work was to identify hyaluronic acid in three food supplements that differ in dosage form and composition. For each sample, a specific isolation procedure for the isolation and concentration of the analyte was proposed. The first step was to remove low molecular weight substances by washing the samples with suitable solvents. The obtained biopolymeric fractions, in addition to hyaluronic acid itself, contained proteins (partially hydrolyzed collagen) as well as various oligo- or polysaccharides such as maltodextrins, starch, and cellulose. The second step was to remove proteins by enzymatic hydrolysis. This step has proven to be very effective in removing collagen that was initially partially hydrolyzed. The separation of cellulose or other water-insoluble components was achieved by dissolving in hot water followed by filtration. Then, hyaluronic acid was precipitated by the addition of copper(II) cations, which form insoluble complexes with anionic polysaccharides. Neutral polysaccharides such as starch and maltodextrins do not form such complexes and remain in solution. The effectiveness of each of these steps was confirmed by Fourier-transform infrared spectroscopy, which is suitable for the structural analysis of organic and some inorganic compounds. The conclusion of this work is the confirmation of the presence of hyaluronic acid in all of the analyzed samples, and the content of this analyte was determined by three analytical methods that gave similar results. On the example of three food additives, the effectiveness of the selected preparative and analytical procedures was demonstrated in assessing the composition of the obtained fractions and quantitative estimation of hyaluronic acid. The approach discussed in this paper can be used to analyze various dietary supplements, as well as medicinal and cosmetic preparations containing hyaluronic acid, for example, as an active substance for the regeneration of cartilage tissue, skin softening or wound healing.

## Figures and Tables

**Table 1:** Composition of food supplements 1–3 declared by manufacturers.

Supplement	Form	Weight (g)	Ingredients	Weight (mg)	Content (% *w*/*w*)
1	Powder doze	13.18	Hydrolyzed collagen	10,000	75.87
			Maltodextrin	2730	20.71
			Sodium	180	1.37
			Vanilla flavor	150	1.14
			Hyaluronic acids	100	0.76
			Sweetener	20	0.15
2	Tablet	0.326	l-Methionine	150	46.01
			Collagen	70	21.47
			Plant extracts	60	18.40
			l-Ascorbic acid	20	6.13
			Hyaluronic acids	10	3.07
			Ubiquinone-10	5	1.53
			Nicotinic acid	4	1.23
			Vitamin E	3	0.92
			β-Carotene	2	0.61
			Zinc	2	0.61
3	Capsule	0.425	l-Ascorbic acid	80	18.82
			Hyaluronic acid	25	5.88
			Vitamin E	6	1.41
			Ubiquinone-10	5	1.18
			Zinc	5	1.18
			Vitamin A	0.8	0.19
			Copper	0.5	0.12

**Table 2:** Yields and composition of the obtained fractions.

Supplement	Fraction	Weight (g)	Yield (% *w*/*w*)	Composition
1 (13.1783 g)	1.1	12.8512	97.50	Biopolymeric substances
	1.2	0.6523	4.95	Polysaccharides
	1.3	0.1013	0.77	Hyaluronic acid
	1.4	0.2861	2.17	Maltodextrins
2 (9.7815 g)	2.1	9.6411	98.56	Biopolymeric substances
	2.2	0.8012	8.19	Cellulose
	2.3	0.3521	3.60	Hyaluronic acid
	2.4	0.1565	1.60	Polysaccharides (starch)
3 (4.2496 g)	3.1	1.1805	27.78	Hyaluronic acid and mineral substances
	3.2	0.0082	0.19	Mineral substances
	3.3	0.2305	5.42	Hyaluronic acid

**Table 3:** Assignment of IR bands of hyaluronic acid for fractions 1.3, 2.3 and 3.3 [38,39,40,41,42,43,44].

Wavelength (cm^−1^)			Vibration Mode	Functional Group, Compound
1.3	2.3	3.3		
3425	3407	3429	ν(OH)	CHOH, CH_2_OH
3277 sh, 3094 sh	3271 sh, 3074 sh	3275 sh, 3098 sh	amide A, B	CONH
2967 sh	2973 sh	2971 sh	ν_as_(CH_3_)	*N*-Ac
2926	2925	2921	ν_as_(CH_2_), ν(CH)	CHOH, CH_2_OH
2878 sh	2876 sh	2883 sh	ν_s_(CH_3_), ν(CH)	*N*-Ac, CHOH
2854 sh	2853 sh	2852 sh	ν_s_(CH_2_), ν(CH)	CHOH, CH_2_OH
2653	2650 sh	2650	ν(OH)	COOH
1738	1735	1745	ν(C=O)	COOH
1657	1658	1657	amide I	CONH
1552	1545	1563	amide II	CONH
1454 sh	1466 sh, 1456 sh	1470 sh, 1450 sh	δ(CH_2_), δ_as_(CH_3_)	CH_2_OH, *N*-Ac
1425	1430, 1413 sh	1430	δ(COH)	COOH
1377	1382	1378	δ_s_(CH_3_); δ(OH)	CH_3_, CHOH
1320	1331	1318	amide III	CONH
1260, 1230 sh	1260 sh, 1231	1261, 1227	amide III, ν(CO)	CONH, COOH
1154, 1126 sh	1154, 1124 sh	1159, 1128 sh	ν(COC)	glycosidic bond
1105 sh, 1079	1106 sh, 1080	1105 sh, 1078	ν(CO)(CC), δ(COH)	pyranoid ring
1044, 1021 sh, 988 sh	1049 sh, 1025, 994 sh	1046, 1022, 988 sh	ν(CO)(CC), δ(COH)	pyranoid ring
947	946	944 sh	γ(CC)_as_	pyranoid ring
923 sh	923 sh	930	γ(COH)	COOH
895	899	896	δ(C1H)	β-anomer
	758	770	ω(C=O)	COOH
699	706	680	γ(CH_2_)	CH_2_OH
608	610	608	skeletal	pyranoid ring
565	577	574	ω(C=O)	CONH
	535		skeletal	pyranoid ring

sh, shoulder.

**Table 4:** Content of hyaluronic acid (% *w*/*w*) in dietary supplements obtained by gravimetry, photometry and isotachophoresis and declared by the manufacturer.

Supplement	Hyaluronic Acid (% *w*/*w*)				
	Declared	Gravimetry	Isotachophoresis	Photometry	
				Mean (*N* = 3)	RSD (%)
1	0.76	0.77	1.00	0.82	2.48
2	3.07	3.60	3.30	3.06	1.36
3	5.88	5.42	5.60	5.96	0.95

**Table 5:** Advantages and disadvantages of analytical methods used in the analysis.

Method	Advantages	Disadvantages
FT-IR spectroscopy	Structure-sensitive	Low sensitivity for quantification
	Non-destructive	Overlapping of the characteristic bands
	Multianalytical	
	Screening of the fractions	
Gravimetry	Rapid and easy	Cannot identify analyte and purity
	No specific equipment	Presence of impurities—overestimation
		Loss of analyte–underestimation
Isotachophoresis	High sensitivity for quantification	Molecular mass/charge ratio of hyaluronic acid will affect the position of zone
	Selective to specific polyanions	
	Multianalytical	May interfere with polyanions
	No interference with uncharged or cationic compounds	
Spectrophotometry	High sensitivity for quantification	Interfere with other compounds
	Easy calibration	Copper(II) oxidation
		Sensitive to pH and ionic strength

## Data Availability

The data presented in this study are available on request from the corresponding author.
